# London Voluntary Hospitals and Their Critics

**Published:** 1892-01-02

**Authors:** 


### The Hospital

A Weekly Journal of

**Science, Medicine, Nursing, and Philanthropy.**

*For Hospitals, Asylums, Infirmaries, Dispensaries, Medical Practitioners, Students, Nurses, and the Charitable Public.*

Vol. XI.—No. 275.] [Jan. 2, 1892.

### LONDON VOLUNTARY HOSPITALS AND THEIR CRITICS.

With a deficiency of £150,000 to face the managers of the voluntary hospitals of London might reasonably experience some depression of spirits. It is necessary, therefore, and in every way desirable at a time of straitened means, such as the hospitals are experiencing to-day, that the managers of these institutions should take counsel together, and, by enlisting the sympathy of the Press, direct public attention to the facts, and continue the agitation until adequate supplies have been poured into their exchequers. Acting in concert with many of the Secretaries of the metropolitan voluntary hospitals, we made a special effort in this direction in our issue of the 19th of December, and to-day we republish with grateful recognition extracts from the many articles which have appeared in our contemporaries in response to our appeal. The £150,000 has still to be raised, but with combined and continuous effort on the part of the representatives of all the hospitals, we have the best reason to believe that in the end this sum at least will be subscribed.

One result of our action has been to elicit a certain amount, we will not say of hostility, but of criticism. The *Pall Mall Gazette* has made itself the mouthpiece of the malcontents. In a leading article on the 26th ult, whilst giving general support to our appeal for funds, it summarised the grounds of attack, and concluded by urging the public and the supporters of hospitals not to give less of their money, but to give more of themselves in personal services to the hospitals. This is excellent advice, which we hope will be widely followed.

The criticisms may be briefly summarised. They are—

(1) That a series of "hospital scandals," whether rightly or wrongly so called, have appeared in newspapers and magazines during the year;

(2) That the authorities wring their hands over this agitation instead of setting to work to remove its cause;

(3) That instead of spontaneously improving the condition of the nurses, hospital authorities have burked enquiry, denied the facts as stated, and arrogantly refused reform;

(4) That we, in these columns, have sneered at and reproached the nurses when victorious in their crusade, calling them "traitors," "querulous ingrates," "creatures whose thoughts were all for self";

(5) That in practice the hospitals are governed by small knots of well-meaning old gentlemen who have a certain amount of time on their hands, but who, taken as a class, are profoundly ignorant of the details of the administration over which they preside, and who do nothing except to take an occasional walk through the wards under the safe conduct of the paid officials, whom they seldom or never venture to contradict.

Before examining these criticisms in detail we must express our satisfaction that the *Pall Mall Gazette* recognises that "in enormous institutions like our hospitals, coping with a mass of difficult work, there must arise mistakes and injustice and defects." This is an important admission, because it ought to convince our contemporary, on a careful examination of the work of our hospitals, that, bearing these difficulties in mind and looking at the question impartially, the voluntary hospitals of London are, as Lord Sandhurst has declared, the most efficient in the world. The hospital authorities frankly recognise the difficulties they have to contend with, and, the *Pall Mall* to the contrary notwithstanding, they have declared and do declare, in season and out of season, that the present arrangements, both for the patients and the nurses, are, for the most part, as comfortable and complete as the money at their command will supply. What the hospitals want is less criticism and more cash. Give the authorities an abundance of money, and, they will be glad to lay it out to the fullest advantage in the interests of the patients and all connected with their administration.

When challenged, the *Pall Mall Gazette* endeavoured to justify its use of the words "Hospital Scandals" by mentioning the Eastern Hospital; the *Truth* campaign against the St. Bartholomew's sanitation, the Glasgow Infirmary, and the London Hospital. Now our readers know, as the *Pall Mall* ought to know, that the Eastern Hospital is a rate-supported and a rate-managed hospital; that St. Bartholomew's is an endowed hospital, which does not appeal in any way to the public for funds; and that the Glasgow Infirmary cannot be mentioned in connection with the *Pall Mall Gazette's* contention, seeing that their original criticisms related entirely to London. In other words, so far as the voluntary hospitals are concerned, on the *Pall Mall Gazette's* own statement, the term "hospital scandal" does not even apply, unless it be in the single case of the London Hospital. Now, it is notorious that the disturbances at the London Hospital have been fomented and continued by a very small clique, some of whom have personal reasons for continuing the agitation. The same people appear at the quarterly courts, time after time, and, despite the fact that their statements have been refuted and their allegations answered over and over again, they continue to repeat them. The time has surely arrived when it is desirable that the authorities and the governors should assert themselves and put a stop to what is becoming a dangerous nuisance. Besides, the criticisms on the London Hospital were made anterior to the issue of "General" Booth's appeal, so that they cannot be held to apply to the present controversy. Mention is made, it is true, of three sensational incidents during the year, but no details are given. From enquiries we have made, we have only been able to identify one such incident, and in that case the authorities punished those responsible for the neglect complained of with commendable promptness. We hope, therefore, that the public will recognise the justice, of coming forward at this juncture in support of our voluntary hospitals, which are not only necessary to the well-being of Londoners as a whole, but from the efficiency of their administration are entitled to receive much more money than is given to them at the present time.

Having disposed of the main charge, we will briefly deal with the others. The wringing of hands theory—and it is nothing but a theory—is laughable in face of the fact that long before it was written the representatives of the principal hospitals had determined to combine, and that they are at the present time actively co-operating to secure by the means suggested by the *Pall Mall Gazette* the necessary funds to do the work entrusted to them.

We should be the last to contend (and we are sure the hospital authorities will agree with us in this) that all hospital committees are perfect. Still the criticisms upon hospital committees in the *Pall Mall Gazette* show that the writer has no accurate knowledge of the system pursued in the administration of the best managed voluntary hospitals. So far from every committeeman being an ignoramus and a noodle, critics will find, if they take the trouble to enquire, that very many of them devote time, influence, hard work, and persistent effort in their endeavours to secure efficient administration for the institutions with which they are connected. There is many a committeeman in active business who shows as much interest in his work for the hospital as he does in his own affairs. House visitors are appointed, whose names are placed upon a *rota*, and they visit every portion of the hospital and the buildings connected with it at regular intervals. Some of these gentlemen arrange to inspect the wards at night, and everything is done to secure the comfort and well-being of the patients, and the happiness and prosperity of the staff.

It is surely time, in the face of such facts as these, to recognise the enormous services which are rendered to every class of Londoners by the voluntary hospitals. Not only do these institutions minister to the needs of the sick Londoner, but they do a great amount of work for the whole country, for the more serious cases are very frequently sent up to the metropolitan hospitals from the country to be dealt with. It is a heavy responsibility to use the influence of an important editorial position to prevent the adequate pecuniary support of a great group of charities doing necessary work which could not be accomplished at all if those charities ceased to exist. We welcome, therefore, the signs of repentance which the *Pall Mall Gazette* exhibits when it urges that "while critics ponder the poor die." We commend this truism to the consideration of all who have the means of supporting the hospitals, and we hope that it is a sentiment which will fructify in the heart of the editor of the *Pall Mall Gazette.* If it does, then for the future, no spicily-headed paragraphs reflecting upon the administration of any reputable hospital will be allowed to appear until some little trouble has been taken to ascertain the truth or falsehood of the statements they may contain. Let us by all means have criticism where criticism is justified by the facts, but until these facts are forthcoming, let us hope, that the giving public will throw off its present waiting attitude and supply the hospitals liberally with the cash they so urgently need.

The question of the nurses is too large a subject to treat in the present article, but we propose to deal with it fully in "The Mirror" next week.

